# *TRPC6* gene promoter polymorphisms in steroid resistant nephrotic syndrome children

**Published:** 2015-02-23

**Authors:** Kempanahalli Basappa Mahesh Kumar, Senguttuvan Prabha, Elumalai Ramprasad, Lakkakula VKS Bhaskar, Periasamy Soundararajan

**Affiliations:** ^1^Department of Nephrology, Sri Ramachandra University, Chennai, India; ^2^Department of Biomedical Sciences, Sri Ramachandra University, Chennai, India; ^3^Sickle Cell Institute Chhattisgarh, Raipur, India

**Keywords:** *TRPC6* gene, End-stage renal disease, Proteinuria, Nephrotic syndrome

## Abstract

**Introduction:** Nephrotic syndrome (NS) is the most frequent cause of proteinuria in children and is emerging as a leading cause of uremia. Among idiopathic NS, 10% of children do not respond to steroids or to any other immunosuppressive therapy, and progress to end-stage renal disease (ESRD). Several studies have investigated the mutations in genes encoding podocyte proteins and their possible associations with several forms of hereditary NS.

**Objectives:** The present study aimed to determine the distribution of the *TRPC6* gene promoter polymorphisms in subjects with features of steroid resistant nephrotic syndrome (SRNS) and controls.

**Patients and Methods:** About 49 unrelated patients with SRNS and 45 age matched controls no renal or other disorders were included in the study. PCR-RFLP was used for genotyping rs3824934 (-254C>G) and rs56134796 (-218C>T) polymorphisms located in *TRPC6* gene promoter region.

**Results:** Both -254C>G and -218C>T are polymorphic in both SRNS patients and controls. No statistically significant differences in genotypes or allele frequencies between SRNS patients and controls were observed. Linkage disequilibrium was not strong and significant and haplotypes were not associated with SRNS. Interaction analysis by multifactor dimensionality reduction (MDR) revealed a significant interaction between -254G>C and -218C>T in <10 years age group.

**Conclusion:** The results demonstrate that the *TRPC6* polymorphisms do not affect susceptibility of SRNS in Indian population. Further replications, preferably a systematic search for *TRPC6* functional variants that affect gene expression are desirable for validation of our findings.

**Implication for health policy/practice/research/medical education:**

To determine the distribution of the TRPC6 gene promoter polymorphisms in subjects with features of steroid resistant nephrotic syndrome (SRNS) and controls, we conducted a study on a total of 49 unrelated patients with SRNS and 45 age matched controls. The results demonstrate that the TRPC6 polymorphisms do not affect susceptibility of SRNS in Indian population. Further replications, preferably a systematic search for TRPC6 functional variants that affect gene expression are desirable for validation of our findings.

## Introduction

Steroid resistant nephrotic syndrome (SRNS) children represent a heterogeneous group characterized by resistance to immunosuppressive drugs and rapid progression to ESRD. Focal segmental glomerulosclerosis (FSGS) is the most common cause of SRNS and accounts for 20% of childhood and 5% of adult cases of ESRD (1). In comparison to minimal change disease (MCD) FSGS is associated with substantial morbidity, and hence elucidation of its pathogenesis has been a focus of research efforts. Over the past one decade, research has been focused on genetic component in pathogenesis of SRNS, in both familial and sporadic cases. Mutations in genes coding for proteins involved in nephron development and an intact podocyte slit diaphragm, are directly involved in autosomal-recessive or dominant forms of FSGS (2-6). The *NPHS1*, *NPHS2*, *WT1* gene mutations are commonly involved in SRNS pathogenesis of Western populations. But their prevalence in Asians is very low. As per our knowledge majority of these gene mutations have not been studied in Indian populations, hence the nature and type of mutations associated with NS is unclear.

*TRPC6* is a transient receptor potential (TRP) channel that plays a role in intracellular calcium signaling and is expressed in a signaling complex with nephrin and podocin in the podocyte slit diaphragm (7). It was hypothesized that glomerular disease is caused by the damaged podocytes through increased *TRPC6* mediated calcium signaling. Malfunction of *TRPC6* leads to a disruption of podocyte cytoskeletal organization, podocyte foot process effacement, glomerular dysfunction, and proteinuric kidney disease. Renal immunofluorescence revealed upregulated expression of *TRPC6* and loss of nephrin in glomeruli. Higher levels of intracellular calcium concentrations were found in the cells expressing mutant *TRPC6* channels compared with cells expressing wild type *TRPC6* (8). This is further supported by the increased glomerular expression of wild type *TRPC6* in acquired forms of proteinuric kidney disease of human and rodents (9). Heterozygous mutations in *TRPC6* were recently identified to cause late onset autosomal dominant FSGS (2,10,11). A recent report documented the presence of *TRPC6* mutations in childhood SRNS, of Turkish children (12).

## Objectives

The present study aimed to determine the distribution of the *TRPC6* gene promoter polymorphisms in subjects with features of SRNS and controls.

## Patients and Methods

### Subjects

Patients with clinical features suggestive of SRNS seen in the nephrology clinic at department of nephrology, Sri Ramachandra University, during the period of March 2012-2014 were included in the study. The existence of consanguinity and the regional birthplace of patients were also noted. A total of 49 unrelated patients with SRNS were recruited in the present study: 18 females and 31 males, with 38 patients (78%) aged less than 10 years. The patients presented with chronic kidney disease with history of SRNS (No remission even after 4 weeks of prednisolone treatment [2 mg/kg/d] or adverse effects related to previous steroid use). Renal biopsies were performed when clinically indicated and detailed clinical history all patients were documented. A total of 45 healthy volunteers with no renal or other disorders matched for age were used as controls. Individuals with secondary glomerulonephritis, previous organ transplantation were excluded from the study. A peripheral blood sample (3 ml) was collected from all the patients and control subjects and genomic DNA was isolated from peripheral blood leukocytes (13).

### Genotyping

Two candidate SNPs in the promoter region of *TRPC6* gene were genotyped using PCR-RFLP. As the GC content of the promoter region (388 base pair fragment) of *TRPC6* is 63.9%, we were able to amplify but not sequence the same, hence we performed RFLP to genotype the known promoter polymorphisms. The sequences flanking to rs3824934 (-254C>G) and rs56134796 (-218C>T) was amplified with the following custom-designed oligonucleotide primers 5’-TTC GCG TCA GCG GCC GAA CT-3’and 5’-TTT GGC TGC TTT CTA GAA CGT CGC T-3’. After initial denaturation (5 minutes at 95°C), 35 cycles of amplification were carried out according to the following scheme: 30 seconds at 95°C for denaturation; 45 seconds at 56°C for primer annealing and 1.5 minutes at 72°C for DNA synthesis. For final elongation, the primers were incubated for 7 minutes at 72°C and then cooled to room temperature. Each reaction was performed in a 25 μl reaction mixture containing 120 ng of genomic DNA (12 µL), 0.15 μM of the primers (1.0 μl forward primer and 1.0 μl reverse primer) and 11 μl 2X PCR master mix. For genotyping the -254C>G, amplified products were digested with AvaI (Fermentas International Inc*.,* Burlington, ON, Canada) at 37°C overnight. The C allele gave four products (194 base pair, 101 base pair, 71 base pair and 22 base pair fragments) whereas the G allele gave three products (194 base pair, 101 base pair and 93 base pair fragments). For genotyping the -218C>T, amplified products were digested with TfiI restriction enzyme (Fermentas International Inc*.*, Burlington, ON, Canada) at 37°C overnight. The presence of a TfiI site (T allele) was indicated by cleavage of the 388 base pair amplified product to yield 2 fragments 251 base pair and 137 base pair. The C allele will not have TfiI site and remain intact (388 base pair). All fragments were electrophoresed on 4% agarose gel (SRL, India), visualized under UV transilluminator.

### Ethical issues

The research followed the tenets of the Declaration of Helsinki. Informed consent was obtained and the research was approved by the Ethics Committee of Sri Ramachandra University, Chennai (Ref: CSP-MED/13/JUN/07/13).

### Statistical analysis

Genotype frequencies were calculated from observed genotypes. Departures of genotype frequencies from Hardy-Weinberg proportions (HWP) for each SNP were tested using chi-square tests with one degree of freedom. The genotype and allele frequencies were also compared using a chi-square test. A *P* value below 0.05 was considered to be significant. Linkage disequilibrium (LD) values of D’ and r^2^ were estimated using Haploview 3.12 (14).

## Results

**Figure 1:**
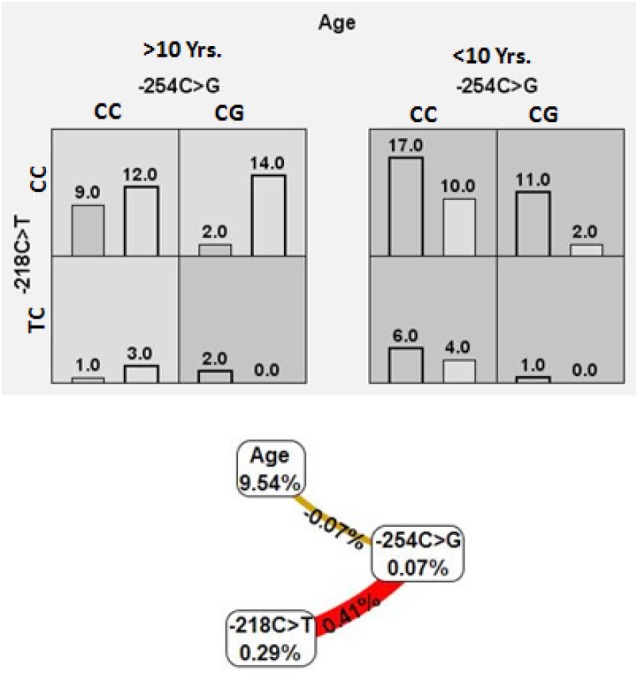
Distribution of high-risk and low-risk genotypes and interaction entropy model. Upper portion of the figure depicted with dark gray and light gray boxes representing highand low-risk factor combinations, respectively. Left bars within each box represented SRNS while the right bars represented control. The heights of the bars are proportional to the sum of samples in each group. Lower portion of the figure depicted with interaction entropy model. Each factor or polymorphism is depicted with its entropy percentage at the bottom, and the percentage presented on each line indicates the interaction percentage of entropy between two factors.

The mean age of the SRNS and control subjects were 7.78±4.12 and 9.49±3.92 years, respectively. The present study is dominated by male subjects, with 63.3% and 73.3% respectively in SRNS and control groups. The prevalence of consanguineous marriage in SRNS patients is about 22.4%. Majority of the NS patients did not consent for biopsy hence renal biopsy was performed on only 16 subjects, among whom 12 patients (24.5%) had minimal change nephropathy (MCN), 4 patients (8.2%) had FSGS. Among SRNS children 84% of them had a serum creatinine of <1 mg/dL. Analysis of two promoter variants (-254C>G: rs3824934; -218C>T: rs56134796) using RFLP revealed that both are polymorphic in the study population with the minor allele frequency of 17.1% and 9% respectively for -254C>G and -218C>T. Both polymorphisms followed Hardy-Weinberg equilibrium (Table 1). The -254C>G (rs3824934) was found in 32.7% of SRNS children and 35.6% controls and their distribution is not significantly different (*P*=0.767). Another promoter polymorphism -218C>T (rs56134796) was found in 20.4% of SRNS and 15.6% of controls and their distribution was also not statistically different (*P*=0.541; Table 1). In addition, the allelic model also showed no significant association between SRNS and controls (Table 1). Linkage disequilibrium between -254C**>**G and -218C>T is not strong and significant with D’ value of 1 and r^2^ value of 0.021. Haplotype analysis is not informative in our study. MDR software was used to analyze the genotypes and age interaction and the results are detailed in Table 2. The best model predicted for 1, 2 and 3 order interactions were not significant. The risk analysis showed the -254G>C (CC) + -218C>T (CC) and -254G>C (CG) + -218C>T (CC) combined genotype of were in high-risk zone in <10 years age group. Where as in ≥10 years age group -254G>C (CG) + -218C>T (TC) combined genotype were in high-risk zone (Figure 1). The entropy graph showed that the independent main effect was largest for age with the information gain value of 9.54%. The -254G>C and -218C>T showed synergistic interaction as indicated by joint information gain value of 0.41%. Besides, the interaction of -254G>C with age was negative, suggesting the redundant effect (Figure 1).

**Table 1:** Distribution of -254C>G and -218C>T polymorphisms in SRNS and controls

**Genotype**	**SRNS**	**Controls**	**P value**
**rs3824934 (-254C>G)**			
CC	33 (67.3)	29 (64.4)	0.767
CG	16 (32.7)	16 (35.6)	
GG	0 (0)	0 (0)	
HWp	0.172	0.147	
C allele	82 (83.7)	74 (82.2)	0.791
G allele	16 (16.3)	16 (17.8)	
**rs56134796 (-218C>T)**			
CC	39 (79.6)	38 (84.4)	0.541
CT	10 (20.4)	7 (15.6)	
TT	0 (0)	0 (0)	
HWp	0.426	0.572	
C allele	88 (89.8)	83 (92.2)	0.562
T allele	10 (10.2)	7 (7.8)	

**Table 2:** Interaction models by MDR analysis

**Best Model**	**Cross Validation Consistency**	**Testing Accuracy**	***P*** ** value**
Age	10/10	0.679	0.270
-254C>G, age	7/10	0.646	0.366
-254C>G, -218C>T, age	10/10	0.659	0.330

## Discussion

Both *TRPC6* Polymorphisms (-254C>G and -218C>T and) were polymorphic and followed Hardy-Weinberg equilibrium. Both -254C>G and -218C>T and genotypes and alleles were not associated with SRNS predisposition. The distribution of -254C>G and -218C>T polymorphisms was similar in MCN, FSGS and non-biopsy NS cases. As these markers separated by only 36 base pairs, linkage disequilibrium was not strong and haplotypes were not associated with SRNS.

So far, several mutations in the *TRPC6* gene have been identified in different ethnic backgrounds. Majority of the studies done in patients with familial FSGS revealed several intronic, synonymous, missense and novel de novo mutations (11,15,16). Many of these mutations were gain of function mutations that resulted in increased calcium current amplitudes (P112Q, M132T, R895C, E897K and Q889K). Some of them did not alter calcium influx (10-17). Only 4 mutations of the *TRPC6* gene have been discovered in sporadic cases of FSGS (8,12,17). *TRPC6* polymorphisms (rs3824935 C/T, rs17096918 C/T, and rs4326755 A/G) did not show statistically significant difference between controls and biopsy-diagnosed membranous glomerulonephritis patients (18). Analysis of 26 patients with primary FSGS and ESRD showed that the mutations in podocyte genes seem to be a rare cause of FSGS and renal failure in German adult patients (19). Comprehensive mutation screening in 64 Czech adult patients with histological proven FSGS/MCD did not find any probable disease-causing mutation in the *TRPC6* gene (20). Screening of 80 FSGS pedigrees with Chinese ancestry revealed presence of a missense mutation (Q889K) in 2 independent families with the mutation rate of 2.5% (21). Analysis of several genes to quantify the contribution of various genes causing FSGS revealed that *TRPC6* related diseases accounted for only 2% (22). By direct sequencing of the entire *TRPC6* gene of the idiopathic membranous glomerulopathy (iMN) cohort identified 13 SNPs, however statistically significant differences in genotypes or allele frequencies between patients and controls were not observed (23).

The frequency of -254C>G and -218C>T in our SRNS patient was 20.4% and 34.0% respectively. Although there are not many studies available to compare our results, *TRPC6*-254C>G promoter variation is associated with enhanced transcription and steroid-resistant NS in Chinese children (18,24). Moreover, the -254C>G SNP creates a binding sequence (GGGGGTCTCC) for nuclear factor-kappa B (NF-κB) an inflammatory transcription factor (25). Functional analyses revealed that the -254C>G SNP enhanced nuclear factor-kappa B-mediated promoter activity and stimulated *TRPC6* expression (25). Inhibition of nuclear factor-kappa-B activity attenuated *TRPC6* expression and decreased agonist-activated Ca2+ influx in pulmonary artery smooth muscle cells of idiopathic pulmonary arterial hypertension patients harboring the -254G allele (25). It was recently demonstrated that the NF-κB negatively regulate the *TRPC6* expression at the gene transcription level in kidney cells (26).

## Conclusion

To our knowledge, this is the first study that has used promoter polymorphisms to study SRNS in a relatively homogenous south Indian population. Our study demonstrated presence of promoter polymorphisms in SRNS patients of Indian origin. Although our study provides statistical evidence for the non-involvement of *TRPC6* promoter polymorphisms in SRNS, our results cannot be considered conclusive. Further replications, preferably a systematic search for *TRPC6* functional variants that affect gene expression are desirable for validation of our findings.

## Limitations of the study

This study has a number of important limitations that needs to be considered. Firstly, coding regions and known mutations were not analyzed, thus comparison of these findings might be limited. Secondly, biopsy was not performed on many of the subjects because of failure to get consent, thus histological correlation might be limited. Lastly, this particular study underutilized the genotyping technology because of high GC content, thus identification of -254C>G homozygous mutants might be limited.

## Authors’ contribution

All authors contributed extensively to the work presented in this paper. PS, SP, and LVKSB supervised the project. MKKB, RE, and LVKSB have carried out the molecular genetic studies, performed statistical calculations and drafted the manuscript. All authors read and approved the final manuscript.

## Conflicts of interest

The authors declared no competing interests.

## Ethical considerations

Ethical issues (including plagiarism, misconduct, data fabrication, falsification, double publication or submission, redundancy) have been completely observed by the authors.

## Funding/Support

This research did not receive any specific grant from any funding agency in the public, commercial or not-for-profit sector.
